# *Pseudomonas* spp. as models for plant-microbe interactions

**DOI:** 10.3389/fpls.2015.00787

**Published:** 2015-09-29

**Authors:** Ramakrishnan Sitaraman

**Affiliations:** Department of Biotechnology, TERI UniversityNew Delhi, India

**Keywords:** *Pseudomonas*, microbiota, phytomicrobiome, plant-microbe interactions, phospholipase, quorum sensing, type VI secretion, lipopeptide

## The phytomicrobiome in context

As is the case with other multicellular eukaryotes, plants are colonized by large numbers of unicellular microorganisms. They may be free-living commensals, epiphytes, symbionts (endophytes), or obligate parasites. The plant holobiont is in effect an ecosystem, and it is of interest to know how this assemblage is established and maintained, and reacts to both biotic and abiotic cues. The current view, initially elaborated in the context of coral-dwelling microbial communites, is that the multicellular organism is more inclusively described by the term “holobiont” that includes associated microbiota, and is a valid unit of natural selection (Rosenberg et al., 2007). The holobiont then, is often dependent on its microbiota for crucial functions, drastic imbalances in which, termed dysbiosis, are thought to lead to compromised or deficient functioning.

The association of plants with microbes is phylogenetically ancient, going back to the macroalgae (Marshall et al., 2006). The role of the microbiota of plants, collectively termed the “phytomicrobiome,” in their overall life cycle is now under investigation, close on the heels of more extensive studies on animal, especially human, microbiota. The development of *Arabidopsis thaliana* (thale cress) and *Brachypodium distachyon* (purple false brome) as model systems for dicotyledonous and monocotyledonous plants respectively, and the availability of genome databases for Pseudomonads (Winsor et al., 2011) and plants (Duvick et al., 2008) indicate that the potential for both hypothesis-based and discovery science are indeed great.

The assembly, development and maintenance of the plant holobiont is not possible without an exchange and sensing of, and responses to, biomolecular cues between its constituents. Within this overall theme, we focus on a few recently discovered, novel inter- and intra-species interactions of some *Pseudomonas* spp., indicating their utility as model systems, and highlighting some previously unforeseen mechanisms that could have a bearing on plant-phytomicrobiome interactions. Note that, for purposes of this article, we use the word “signaling” to refer generically to the sensing and response of organisms to environmental cues of both biotic and abiotic origins.

## Some aspects of the social biology of *pseudomonas* spp.

The genus *Pseudomonas* is the most numerous among the cataloged genera of Gram-negative bacteria (Gomila et al., 2015). The ubiquity and metabolic versatility of this genus allows it to colonize a wide range of natural habitats and adopt a variety of lifestyles. *Pseudomonas* spp. have been isolated from each of the ecological niches within a plant as stated earlier (for a compilation, see Table 1 of Mercado-Blanco and Bakker, 2007). Their known ability to interact with and influence other bacteria, fungi, and multicellular organisms in a variety of biological contexts, and the availability of experimental tools for their genetic manipulation, should greatly facilitate the translation of knowledge for a wide range of practical applications.

At the outset, it is worth recalling that strains of *P. putida* and *P. aeruginosa* were the first living, genetically modified organisms to be patented for a specific application (biodegradation of petrochemicals—camphor, octane, salicylate, and naphthalene)—truly heralding the modern era of genetically modified organisms (Chakrabarty, 1981). The genus *Pseudomonas* is behaviorally very versatile, with free-living as well as parasitic forms capable of colonizing a wide variety of host organisms and ecological niches within hosts. For example, *P. aeruginosa* (PA) is a free-living soil bacterium that is also an opportunistic pathogen of both plants and animals, *P. syringiae* (PS) is an opportunistic plant pathogen, *P. putida* (PP) has been extensively used in bioremediation for its ability to utilize a wide range of hydrocarbons as carbon sources, and both *P. putida* and *P. fluorescens* (PF) are promising growth-promoting and biocontrol agents.

In order to understand the role of the bacterial component of the phytomicrobiome in plant physiology, the functional analysis of bacteria colonizing multiple ecological niches provided by the plant—the roots (rhizosphere), leaves (phyllosphere), surfaces (ectosphere), and tissues (endosphere)—needs to be undertaken, ideally *in situ* and over the several developmental stages of the plant. This is an understandably formidable task, and the utility of a model bacterium in this context is apparent. From a bacterial viewpoint, it has to sense the presence of, and stimuli from, potential hosts as well as competitors (of the same or different species), strategize in a manner that allows it to reach the host, survive competition, and colonize, gain access to resources, and persist for a reasonable length of time in the face of perturbations. The establishment and resilience of the plant-microbe interaction is therefore dependent on the exchange and sensing of a variety of signals by both types of partners, often simultaneously, and combinatorially.

### Bacterial quorum sensing and inter-species competition

*Pseudomonas* spp. possess quorum-sensing (QS) systems that synthesize and sense hormone-like messages of diverse origins in their immediate environment. QS systems are often linked with other regulons, leading to different phenotypes (for a review, see Venturi, 2006). For example, PP produces cyclic lipopeptide surfactants putisolvin I and II, that are under the control of QS and disrupt biofilms (Kuiper et al., 2004; Dubern et al., 2006). Interestingly, this can happen not only at the stationary phase, but also stochastically in the early stages of growth resulting in swarming motility (Cárcamo-Oyarce et al., 2015), promoting colonization of fresh surfaces. Other putisolvin-like lipopeptides of PP have been found to exhibit lytic activity against the zoospores of the fungal pathogen *Phytophthora capsici* zoospores *in vitro*, inhibit growth of the fungal pathogens *Botrytis cinerea* and *Rhizoctonia solani* in addition to being involved in swarming motility (Kruijt et al., 2009). In more general terms such interactions could contribute to the overall composition of the phytomicrobiome by modifying its diversity, and contribute to its resilience to perturbation by invaders.

Plant growth promotion effects of *Pseudomonas* spp. may also be under QS control, as was demonstrated in the case of QS-controlled production of an *N*-acyl-L-homoserine lactone (AHL), cyclic dipeptides and their derivative diketopiperazines (DKPs) by PA. Exposure of *A. thaliana* seedlings to 3-oxo-C12-AHL produced by the LasI AHL synthase causes growth inhibition of the primary root, while DKP stimulated the growth of lateral roots (Ortiz-Castro et al., 2011). The presence of orphan AHL transcriptional regulators such as QscR in PA that lack a cognate AHL synthase and bind with relaxed specificity to both endogenously and exogenously produced AHLs adds another layer of complexity to plant-phytomicrobiota interactions (for a recent and detailed review, see Chugani and Greenberg, 2014). Likewise, pseudomonads as well as other plant-associated bacteria have been found to encode a unique family of orphan (or solo) AHL transcriptional regulators that are uniquely responsive to unknown plant and/or bacterial signal molecules (Patel et al., 2013).

That one component of the microbiota may influence another indirectly by modulating host signals and responses has been dramatically demonstrated recently in both plant and animal contexts. PS pathovar tomato (PSt) infection of *Arabidoposis thaliana* leaves induces the plant enzyme phospholipase Db1 (PLDb1) that is a negative regulator of the salicylic acid-dependent resistance to PS, but is a positive regulator of the jasmonic acid-dependent resistance to the fungal pathogen *Botrytis cinerea*. Even more interestingly, infection with an avirulent PSt strain that expresses the effector AvrRpt2 secreted by the type III secretion system can also lead to resistance against virulent Pst that does not express AvrRpt2 (Zhao et al., 2013). Thus, indirect microbial modulation of the host can cause subtle, even strain-level, shifts in the composition of microbiota, depending on the temporal sequence of host colonization. In what may well be a case of convergent survival strategies, PA infection of airways in human patients of cystic fibrosis induces airway cells of the airway epithelium to produce secretory phosopholipase A2, which is bactericidal for Gram-positive bacteria such as *Staphylococcus aureus* but relatively less so for PA (Pernet et al., 2014). This effectively allows PA to proliferate at the expense of *S. aureus*.

### Identification of putative type VI effectors in plant-associated *pseudomonas* spp.

The ability of PA to infect both plant and animal hosts, and the identification of a common set of virulence determinants during plant and animal infections (Rahme et al., 1997, 2000), along with genome sequence information can be exploited to identify potential effectors and predict putative mechanisms of interaction with the host plant in the context of other *Pseudomonas* spp. Recent, extensive analyses of the *A. thaliana*-associated microbiota indicate that *Pseudomonas* spp. are preferentially enriched in the endophytic compartment of the plant, as compared to the rhizosphere (Bulgarelli et al., 2012; Lundberg et al., 2012). Therefore, the identification of conserved effectors within the pseudomonad lineage can be used as a starting point to probe plant-microbe interactions. The type VI secretion systems (T6SS) merit special attention in this regard as they are widespread among diverse Gram-negative bacteria, both pathogenic and non-pathogenic including *Pseudomonas* spp. (Barret et al., 2011), and can potentially deploy effectors targeting both prokaryotic and eukaryotic cells (Jiang et al., 2014). Two effectors secreted by T6SS in PA that are known to target host cells, and their orthologs identified in plant-associated *Pseudomonas* spp. are listed in Table 1. The functionality of these effectors on plant cells, if verified, can provide important information about the assembly and disruption of bacterial communities, as well as their interaction with the host plant.

**Table 1:** **A representative list of putative effectors potentially targeting plant cells and encoded by T6SS in plant-associated *Pseudomonas* spp**.

**Effector molecule(s) of *P. aeruginosa***	**Locus tag/Strain of PA**	**Known function and context in PA**	**Reference(s) for known functions**	**Plant-associated *Pseudomonas* spp**.	**Ortholog locus tag**
Phospholipase D (PldB)	PA5089/PAO1	Encoded by the H3-T6SS. Elimination of compteting bacteria. Promoting PA internalization by host (human) epithelial cells	Jiang et al., 2014	*Pseudomonas* sp. UW4	PputUW4_03278
				*P. syringae* pv. *Phaseolicola* 1448A (pathogen)	PSPPH_0117
				*P. syringae* pv. *Syringae* B728a (pathogen)	Psyr_4970
Valine-glycine repeat protein (VgrG2b)	PA0262/PAO1	Encoded by the H2-T6SS. Delivered into host (human) epithelial cells, promotes microtubule-mediated PA internalization by direct interaction with microtubules	Sana et al., 2015	*P. syringae* pv. *syringae* B728a	Psyr_4080
				*Pseudomonas* sp. UW4	PputUW4_03083
				*P. putida* F1 (orthologs also present in strains HB3267, KT2440, H8234, ND6, GB-1, NBRC 14164, W619 and DOT-T1E)	Pput_2117

*These have been identified based on two T6SS effectors in P. aeruginosa, PldB and VgrG2b, that are known to target eukaryotic host cells. Orthologs were identified by searching the Pseudomonas database (http://beta.pseudomonas.com); (Winsor et al., 2011) and the Pseudomonas ortholog database (http://pseudoluge.pseudomonas.com/pseudoluge/named/list/search?field=locus_tag&value=PCHL3084_RS00035); (Whiteside et al., 2013)*.

## Conclusions and future directions

The foregoing account suggests new lines of inquiry into the signals that drive the formation and maintenance of the plant microbiota. Can systemic effects on the host and/or microbiota be mediated by diffusible signals produced in one part of the plant? If so, over what distances do these effects extend, and how are they mediated? What is the role of conserved and functional T6SS effectors in diverse plant-bacteria associations that range from commensalism to symbiosis? In the effort to understand the relative contribution of different components of the microbiota to the plant holobiont, it may be remembered that abundance alone may not truly reflect the relative importance of the species/strain in question. Numerically less abundant species could be key players within the microbiota, assuming the role of “keystone” species, as has been suggested earlier (Saraswati and Sitaraman, 2014).

A potential limitation in reliance on Gram-negative pseudomonads as model systems is that their relative importance may depend on environmental conditions. For example, *Pseudomonas* spp. may be an important disease-suppressive agent in a moist and temperate environment in the Netherlands (Mendes et al., 2011), whereas the Gram-positive *Bacillus* spp. contribute to disease suppression in Egypt, a more arid zone (Köberl et al., 2011). Over reliance on *Pseudomonas* spp. as models could therefore potentially overlook unique interactions and mechanisms operative over large geographical areas and ecological zones. Also to be remembered is that most studies of microbiota (plant or animal) focus on the bacterial component alone, and the role of fungi and archaea is less studied and understood.

The microbiota of multicellular organisms, whether plant or animal, present a case wherein simultaneous and combinatorial interactions have to be identified, and their relative importance determined. To this end, the identification of effectors and the delineation of mechanisms of interaction are required. The predictive and inferential value of *Pseudomonas* spp.-based models that can be probed with conventional as well as high-throughput methods is therefore undeniable, and insights so gained have immense potential to inform and refine our efforts to dissect the mechanistic bases of interactions taking place in the plant holobiont.

### Conflict of interest statement

The author declares that the research was conducted in the absence of any commercial or financial relationships that could be construed as a potential conflict of interest.
